# Multi-Dimensional Assessment of Eating Behaviour in the Hungarian Adult Population: Motivations, Health–Taste Attitudes, and Eating Styles Using TEMS, HTAS, and TFEQ-R21

**DOI:** 10.3390/nu18213690

**Published:** 2026-11-07

**Authors:** Petra Platz, Márk Szakály, Kinga Lili Balogh, Péter Huszka

**Affiliations:** 1 Kautz Gyula Faculty of Economics, Széchenyi István University, 9026 Győr, Hungaryhuszkap@sze.hu (P.H.); 2 Faculty of Economics and Business, University of Debrecen, 4032 Debrecen, Hungary; szakalymark@proton.me

**Keywords:** eating behaviour, public health nutrition, TFEQ-R21, TEMS, HTAS, Hungary, functional foods, eating motivations, health attitudes, consumer segmentation

## Abstract

Background/Objectives: Eating behaviour patterns are fundamental determinants of chronic disease risk and public health. Hungary faces among the highest rates of obesity, cardiovascular disease, and diet-related mortality in Europe. Despite this, population-level consumer profiling based on validated multi-scale instruments remains limited. This study used nationally representative samples to assess eating motivations, health and taste attitudes, and eating styles among Hungarian adults, and grouped adults into consumer segments for each measure. Methods: Two independent nationally representative cross-sectional surveys were conducted: (1) eating attitudes (TFEQ-R21) and health/taste attitude scales (HTAS) were assessed in 2021 (n = 1000); (2) eating motivations (TEMS) were assessed in late 2019 (n = 1000). Exploratory factor analysis (EFA) was applied to each instrument, and confirmatory factor analysis (CFA) to the TFEQ-R21 and to both HTAS subscales. Consumer segmentation was performed using hierarchical and K-means cluster analysis. Sociodemographic differences across segments were explored via chi-square tests and one-way analysis of variance (ANOVA). The perceived palatability and healthiness of functional food categories were examined across TFEQ-R21 segments. Results: The TEMS yielded eight eating motivation factors and five consumer segments: Traditional Simplifiers (17.9%), Frugal Social and Emotional Eaters (19.9%), Balanced Self-Rewarders (27.5%), Health-Conscious (18.3%), and Indifferent (16.4%). The HTAS health subscale produced four clusters, with Health-Conscious Metropolitans (19.8%) as the primary functional food target. The HTAS taste subscale identified taste-oriented consumers (26.9%) alongside three further groups. The TFEQ-R21 confirmed a three-factor eating attitude structure (emotional eating, uncontrolled eating, cognitive restriction; explained variance 57.2%) and yielded five behavioural clusters. Controlled Dieters represented 20.6% of the sample; the other TFEQ-R21 segments showed average, uncontrolled, or emotionally driven eating profiles. Perceived palatability and healthiness of functional foods differed across TFEQ-R21 segments (one-way ANOVA, unadjusted *p* < 0.05 in six of ten categories for palatability and eight of ten for healthiness), and Controlled Dieters gave the highest mean rating in eight of ten categories on each measure. Conclusions: The parallel application of three validated eating behaviour scales describes eating attitudes, motivations and consumer segments among Hungarian adults. Habitual, taste-oriented and average-profile segments were common, and each instrument also yielded a smaller health-oriented segment (about 18–21%). These cross-sectional findings on attitudes and self-reported motivations may inform segment-specific communication, which should be tested in future intervention studies.

## 1. Introduction

The relationship between dietary patterns and non-communicable chronic diseases is well established. Globally, poor diet is a leading risk factor for premature death and disease, and obesity is itself a major determinant of type 2 diabetes, cardiovascular disease, hypertension, and certain cancers [1,2]. Worldwide, an estimated 1.9 billion adults are overweight or obese and about 2 billion people are affected by micronutrient deficiencies, while an estimated 11–26 million people in Europe suffer from food allergies or intolerances [3].

Hungary presents a particularly critical case. Among OECD member countries, Hungary ranks among the highest in obesity prevalence for both men and women. Overweight affects approximately 40% of men and 32% of women, with obesity rates approaching 32% in both sexes [4]. Diet-related non-communicable diseases account for a disproportionately high share of premature mortality in the country. Research has consistently shown that 25–70% of chronic conditions are preventable through dietary modification, including more than 30% of cardiovascular disease burden and more than 35% of malignant tumours [5,6].

In response, researchers and food manufacturers have paid growing attention to functional foods: foods with one or more proven health benefits beyond basic nutrition [7]. However, consumer acceptance of these foods varies widely and depends on individual differences in health attitudes, taste preferences, and eating motivations [8,9]. Effective product positioning, health communication, and dietary intervention require a thorough understanding of population-level eating behaviour profiles.

Despite this recognised need, population-wide multi-scale eating behaviour profiling has remained rare. The majority of published studies apply a single validated instrument to non-representative or clinical convenience samples [10,11]. To our knowledge, the three instruments have not previously been reported together for a nationally representative Hungarian sample, although the TFEQ-R21 [12] and the TEMS [13] have each been analysed separately in representative Hungarian samples, and multi-instrument studies remain rare worldwide.

This study addresses that gap using three validated instruments on two nationally representative samples of Hungarian adults: (1) the Three-Factor Eating Questionnaire (TFEQ-R21), which measures emotional eating, uncontrolled eating, and cognitive restriction; (2) the Health and Taste Attitude Scales (HTAS), which measure how much consumers value health versus taste in food choice; and (3) the Eating Motivation Survey (TEMS; 45-item version, see Section 2.2), which covers the range of reasons behind food choices. To our knowledge, this is the first paper to report all three instruments together and to compare how TFEQ-R21 segments perceive functional food categories. Parts of the data have been analysed before [12,13,14]; the new elements are the HTAS analyses, the functional food comparison, and the joint discussion of the three instruments.

We tested four hypotheses.

**H1:**  *The 15-factor structure of the original TEMS [15] will not be fully reproduced in a Hungarian sample, in line with cross-cultural findings [16,17].*

**H2:**  *A distinct health-oriented motivational segment can be identified among the TEMS segments, as in earlier segmentation studies [18,19,20].*

**H3:**  *The three-factor structure of the TFEQ-R21 is supported in a reduced 16-item version, as reported by Szakály et al. [12].*

**H4:**  *A controlled-eating segment with high cognitive restraint and low emotional eating emerges from the TFEQ-R21 segmentation [21,22,23].*

We also ask how TFEQ-R21 segments differ in the perceived palatability and healthiness of functional food categories (RQ1). Because H1–H4 were formulated with knowledge of earlier analyses of these data [12,13], they are replication checks and not independent a priori tests.

### 1.1. The TFEQ-R21 and Eating Styles

The Three-Factor Eating Questionnaire, originally developed by Stunkard and Messick [24] and revised to its 21-item version by Karlsson et al. [25], is one of the most widely applied measures of eating attitudes. The revised instrument (TFEQ-R21) captures three distinct factors: emotional eating (eating in response to negative affect), uncontrolled eating (inability to restrain intake in the presence of food cues), and cognitive restriction (deliberate limitation of food intake). Its three-factor structure has been replicated across diverse populations [25,26,27], including a Hungarian general population adaptation by Czeglédi and Urbán [28]. Szakály et al. [12] rejected the original 21-item model in a representative Hungarian sample (n = 1000) and validated a 16-item version (TFEQ-R16), which is the model examined here.

Cluster analysis based on TFEQ factors has been applied to identify distinct eating behaviour profiles [21,22,23]. The number of emerging segments has ranged from three [21,22] to five [23], with typical labels identifying controlled, indifferent, emotionally driven, and uncontrolled eater groups. Hungarian segmentation studies based on the TFEQ have been few: Szabó et al. [23] validated 12 of the 21 items (six emotional, three uncontrolled, and three cognitive restraint items) and identified five segments. TFEQ-R21 studies still link eating style subscales—especially emotional eating—to wider physical and mental health outcomes. This shows the instrument’s continued relevance to nutrition and public health research [29]. Similar research in Azerbaijan found different eating style patterns among young women, shaped partly by cultural and religious food norms [30]. This shows that eating style patterns need to be studied in each population, rather than assumed to be the same everywhere.

### 1.2. The HTAS: Health Versus Taste Orientation

The Health and Taste Attitude Scales (HTAS) were developed to capture the relative importance consumers place on the healthiness versus the taste of foods [31]. The health subscale measures interest in nutritional quality, avoiding additives, and functional food properties. The taste subscale measures enjoyment, sensory appeal, and indulgence. Roininen et al. [31] demonstrated adequate reliability and validity across Finnish and British samples. Later HTAS studies replicated its structure in further countries and used the health subscales to derive consumer clusters that differed in interest in food-related health [18,19]. In a large, representative sample of Polish girls and young women, positive health-related HTAS attitudes were linked to diets rich in fruit and vegetables. Negative attitudes toward natural products and stronger taste attitudes predicted a less healthy diet [32]. In Hungary, consumer segmentation based on healthy eating has also found groups ranging from strongly health-driven to indifferent [20]. This matches the health-versus-indifference divide seen in the present HTAS clusters.

### 1.3. The TEMS: Eating Motivation Survey

The Eating Motivation Survey (TEMS) by Renner et al. [15], based on earlier work by Steptoe et al. [33], provides a comprehensive taxonomy of reasons behind food choice. The full instrument comprises 15 primary factors, including food enjoyment, habit, health, naturalness, price, social environment, self-reward, and emotional regulation. The original German sample yielded 15 clearly separated factors, but studies in other cultures have found different factor structures [16,17], reflecting culturally specific eating motivations. Szakály and Soós [13] have previously applied the TEMS to a representative Hungarian sample and reported eight factors and five consumer segments; the present paper re-reports and extends these results using the same dataset. The TEMS health motive subscale has been used on its own elsewhere. Among older German adults living independently, stronger health-oriented eating motivation was linked to eating more plant-based protein, though not to total protein intake [34]. In a sample of Polish adults surveyed during the COVID-19 pandemic, TEMS eating motives—especially affect regulation—together with pandemic-related stress significantly predicted emotional overeating [35].

### 1.4. Functional Food Acceptance and Eating Behaviour Profiles

Consumer acceptance of functional foods depends on health consciousness, product-specific beliefs, and general eating attitudes [8,9,36]. Health-conscious consumers demonstrate significantly higher willingness to try and purchase functional food products and perceive them as both tastier and healthier than less health-conscious peers [37]. Personalised nutrition communication based on behavioural segmentation has been shown to make dietary advice more effective [38]. Segmentation approaches that link individual traits and values directly to functional food consumption have also been used in other countries [39]. Understanding the functional food perception of different eating behaviour segments is, therefore, both theoretically relevant and commercially actionable.

## 2. Materials and Methods

### 2.1. Study Design and Participants

Two independent surveys were conducted. The first (TFEQ-R21 and HTAS) was carried out in 2021 (during the COVID-19 pandemic) using a nationwide face-to-face questionnaire administered to a proportional quota sample of 1000 Hungarian adults (aged ≥18 years). Regional quotas followed the structure set by the Hungarian Central Statistical Office (HCSO). Settlements within regions were selected using simple random sampling. Households within settlements were reached by random walk; within each household, the respondent aged 18 or older whose birthday fell nearest to the survey date (the birthday key method) was chosen to randomise selection within the household. Because the random sample over-represented women and older respondents, multi-dimensional weights for region, settlement type, sex, and age were applied; the weighted sample deviates from the HCSO population by at most 2.4 percentage points on these four dimensions (Supplementary Table S1).

The second survey (TEMS) used the same random walk and birthday key procedures, with personal interviews at the end of 2019 and data entry in 2020, and yielded a cleaned sample of n = 1000. It, therefore, preceded the COVID-19 pandemic in Hungary (first cases were confirmed in March 2020), whereas Survey 1 took place during it. The sample was reported as representative for sex and age group. No personal identifying data were collected.

Data provenance. The data analysed here derive from the doctoral dissertation of M.SZ. (University of Debrecen, 2025) [14] and have been partly analysed before: the TFEQ validation in [12], the TEMS factor structure and segments in [13], and all three instruments in the dissertation. The present paper re-reports these results where needed for comparison and adds the HTAS analyses, the functional food comparison, and the cross-instrument discussion. 

### 2.2. Instruments

The Hungarian TFEQ-R21 [28] was administered in full (21 items). Five items (on avoiding the stockpiling of tempting foods, deliberately eating less, hunger and the degree of self-restriction; four on 4-point scales and one on the 8-point scale of the original) were removed after EFA because of cross-loadings under maximum likelihood extraction and uninterpretable components under PCA, and a CFA of the full model confirmed these problems (Section 2.3). The resulting 16-item model (TFEQ-R16) is the one validated by Szakály et al. [12]. Items were rated on a 4-point Likert scale (1 = ‘definitely true of me’ to 4 = ‘not at all true of me’); for analysis and reporting, responses were reversed so that higher scores indicate stronger endorsement. The HTAS [31] comprised 20 health attitude items and 18 taste attitude items, which were formulated by M.SZ. for this study on the basis of the HTAS framework (no separately validated Hungarian translation of the original scale was used), and each was rated on a 7-point Likert scale (1 = ‘strongly disagree’; 7 = ‘fully agree’). The TEMS comprised 45 items (the dissertation does not state which items of the full instrument were omitted) rated on a 7-point frequency-of-motivation scale (1 = ‘never motivates’; 7 = ‘always motivates’).

Sociodemographic variables collected in all surveys included gender, age, highest completed education, marital status, legal/employment status, net monthly income, self-rated health consciousness, and body mass index (BMI) computed from self-reported height and weight.

Functional food perception. Respondents rated the perceived palatability (1 = not at all tasty; 5 = very tasty) and the perceived healthiness (1 = not at all healthy; 5 = very healthy) of 10 functional food categories: vitamin-enriched products (e.g., muesli, soft drinks); mineral-enriched products (e.g., calcium-enriched cheese, milk); probiotic products (e.g., yogurt, kefir, margarine); low-sugar or sugar-free foods; low-fat foods; lactose-free products; high-fibre foods (e.g., fibre-enriched drinks); gluten-free foods (e.g., bread); protein-enriched products (e.g., energy bars); and low-sodium foods.

### 2.3. Statistical Analyses

For the TFEQ-R21, initial exploratory factor analysis (EFA) using maximum likelihood extraction was performed. Five items exhibiting excessive cross-loadings were removed, and EFA was re-run on the 16-item solution. Reliability was assessed by Cronbach’s α and, for the HTAS and TFEQ-R21, also by composite reliability (CR). Confirmatory factor analysis (CFA) was executed in R version 3.5.0 (R Foundation for Statistical Computing, Vienna, Austria) using RStudio (Posit PBC, Boston, MA, USA; https://posit.co/products/open-source/rstudio). Error covariances between items of the same latent variable were allowed where modification indices were large. Model fit was assessed using χ^2^/*df*, CFI, TLI, NFI, and RMSEA, interpreted against common cut-offs (e.g., Hu and Bentler [40]). The same CFA procedure was applied to the HTAS health and taste factors. EFA used maximum likelihood extraction with Varimax rotation. EFA and CFA were run on the same sample and error covariances were freed, so the fit indices are likely to be optimistic. All further analyses were conducted in IBM SPSS Statistics, version 23.0 (IBM Corp., Armonk, NY, USA).

For segmentation in all three models, factor scores were computed by principal component analysis run separately on the items of each latent factor (to avoid cross-loadings) and used as cluster-forming criteria. Hierarchical cluster analysis (Ward’s method, squared Euclidean distance) was used to determine the optimal number of segments. K-means cluster analysis was then run using the resulting cluster centres. One-way ANOVA on the clustering variables was used only to describe which factors differentiated the clusters; because the clusters were formed on these variables, significance is expected and is not evidence of validity, and no further stability checks were carried out. Cluster analyses excluded cases listwise (SPSS), giving n = 979, 941 and 976. Segment labels summarise cluster centroids; ‘Indifferent’ denotes a profile close to the sample mean on all factors, not a measured lack of interest. Between-cluster sociodemographic differences were evaluated with cross-tabulations and simple hypothesis tests; statements on over- or under-representation compare a group’s share within a segment with its share in the sample, and test statistics for individual profile statements are not available. Post hoc comparisons for perceived palatability and healthiness of functional food categories used one-way ANOVA for each of the 10 categories across the five TFEQ-R21 segments (uncorrected *p*-values; with 20 tests, a Bonferroni threshold would be *p* < 0.0025). Pairwise post hoc tests, effect sizes, and covariate-adjusted models were not available, so these comparisons are descriptive.

## 3. Results

### 3.1. TEMS: Eating Motivations

#### 3.1.1. Descriptive Statistics

Table 1 presents the top 10 eating motivation statements from the 45-item TEMS. The highest-ranked motivations were hedonic and habitual in nature. Food love (‘because I love it’; M = 6.01, SD = 1.36), taste (‘because it tastes good’; M = 5.87), and familiarity (‘because I know it well’; M = 5.82) occupied the top three positions. Physiological hunger (M = 5.74) ranked fourth, followed by habitual choices. All top-10 items showed negative skewness, indicating that most respondents considered these motivations self-applicable.

The 10 least important motivations (Table 2) were dominated by social compliance (e.g., ‘because they expect me to eat it’), desire to impress others, and emotional dysregulation (loneliness, sadness, disappointment). All had means of about 3.0 or lower on the 7-point scale (Table 2), and all showed positive skewness, indicating the majority considered them non-applicable.

#### 3.1.2. Factor Structure

EFA yielded eight factors. Because 45 items were administered, and not the full instrument, the smaller number of factors may partly reflect the reduced item set. This differed from the 15-factor original German TEMS model [15] and from TEMS results in other cultures [16,17], consistent with the cross-cultural variation reported in the literature. The factors were labelled: (1) Social Anxiety (combining social norms, social image, and anxiety-driven motivation; Cronbach’s α = 0.916); (2) Comfortable Enjoyment (convenience and food familiarity; α = 0.876); (3) Health Awareness and Weight Control (natural ingredients, health motivation, weight-related cues; α = 0.856); (4) Price Sensitivity (all price-related items; α = 0.782); (5) Social Eating (voluntary prosocial orientation; α = 0.800); (6) Self-Reward (self-indulgence; α = 0.731); (7) Habit- and Tradition-Driven Eating (customs and routines; α = 0.637); and (8) Seductive Nature of Food (visual and sensory appeal; α = 0.770). Full reliability statistics are reported in Section 3.4.

#### 3.1.3. Cluster Analysis

K-means cluster analysis yielded five significantly differentiated segments (F-test; *p* < 0.001). Cluster sizes and proportions are shown in Table 3.

Traditional Simplifiers (17.9%): Characterised by comfort, familiarity, habitual patterns, and food love as primary motivations. All items from the habit/tradition and convenience factors produced high cluster-specific means. Mild price sensitivity was also present. Members appear to be guided primarily by established routine rather than active food choice.

Frugal Social and Emotional Eaters (19.9%): A distinctive mix of proactive social motivation (‘eating is a social event’) and anxious social conformity (‘it would not be polite not to eat it’), combined with strong emotional regulation eating (loneliness, sadness, disappointment) and high price sensitivity. Visual attractiveness and social image also characterised this group.

Balanced Self-Rewarders (27.5%): The largest cluster, defined by self-reward as the primary driver, but with equal representation of tradition, naturalness, food love, physiological need, and health motivation (each contributing approximately 15% of high-mean items). This group illustrates a balanced motivational profile with hedonism as its core.

Health-Conscious (18.3%): All weight-control items (‘because it is low-calorie’, ‘because I watch my weight’, ‘because it is low in fat’) were high-ranking, as were explicit health motivation items (‘because I think it is healthy’, ‘because it keeps me in shape’). Social, emotional, and indulgent motivations were underrepresented.

Indifferent (16.4%): No single statement achieved a high mean in this cluster. Proportional analysis suggested some influence of habit, tradition, convenience, and physiological need, but no motivation was strongly endorsed. This cluster represents consumers for whom food choice is largely automatic and low-involvement.

### 3.2. HTAS: Health and Taste Attitude Scales

#### 3.2.1. HTAS Health Subscale: Factor Analysis and Cluster Solution

EFA (maximum likelihood, Varimax rotation) of the 20 health attitude items (three items removed because they loaded on two factors, leaving 17) yielded four factors explaining 48.3% of the total variance (Kaiser–Meyer–Olkin [KMO] measure of sampling adequacy = 0.842; Bartlett’s *p* < 0.001): (1) Health Consciousness (seven items; explained variance 23.9%; α = 0.837; CR = 0.878), including attention to vitamins and minerals, avoidance of additives, and adherence to a balanced diet; (2) Health Risk Behaviour (four items; 13.4%; α = 0.807; CR = 0.782), comprising avoidance of cholesterol-raising foods and low sensitivity to food health risks; (3) Sceptical Disbelief (three items; 6.5%; α = 0.705; CR = 0.581), encompassing doubt about the health superiority of organic or reduced-energy products and indifference to E-numbers; and (4) Belief in Energy Reduction (three items; 4.6%; α = 0.738; CR = 0.355), reflecting confidence that energy-reduced products preserve weight and cholesterol control. CFA fit indices are given in Section 3.3.2.

Four consumer clusters were identified from the health subscale factors (Table 4).

Misguided Consumers (30.4%): The largest group. These consumers selectively engage with some health-motivated behaviours (e.g., avoiding processed foods) but show scepticism toward the health benefits of organic food, functional products, and additives. They are overrepresented in smaller towns and among lower-educated respondents.

Health-Conscious Metropolitans (19.8%): Strongly health-oriented consumers who strive for a balanced, low-fat diet enriched with vitamins and minerals, regularly consume organic produce, and actively avoid additives. Women and higher-income groups are overrepresented. This group constitutes the primary target for functional food marketing.

Health Risk Takers (23.7%): These consumers least consistently avoid foods that raise blood cholesterol, are minimally influenced by food healthiness in their choices, and do not concern themselves with health risk. Men and older respondents dominate; they report average or weaker health consciousness.

Indifferent (26.1%): No notable endorsement of any health belief dimension. This group shows no outlier on any subscale, suggesting near-complete disengagement from health-based food reasoning. Active manual workers predominate, with overrepresentation in Central and Western Transdanubia.

#### 3.2.2. HTAS Taste Subscale: Factor Analysis and Cluster Solution

EFA (maximum likelihood, Varimax rotation) of the 18 taste attitude items (two items removed because they loaded on two factors) yielded five factors explaining 57.8% of the variance (KMO = 0.797; Bartlett’s *p* < 0.001): Craving (three items; α = 0.956; CR = 0.745), Self-Reward (three items; α = 0.843; CR = 0.688), Wanting (three items; α = 0.870; CR = 0.613), Palatability (three items; α = 0.802; CR = 0.617), and Avoidance of Self-Indulgence (four items; α = 0.580; CR = 0.366). The last factor has low reliability and is interpreted cautiously. CFA fit for the taste scale: χ^2^ = 1224.99, *df* = 94, CFI = 0.992, TLI = 0.990, and RMSEA = 0.032. The Taste subscale cluster analysis similarly produced four distinct segments: Conscious Older Adults (23.8%), who rejected emotional eating and food as pleasure; Controversial Middle-Aged (29.0%), characterised by contradictory behaviour (simultaneous endorsement and rejection of comfort eating); Indifferent (20.4%), reporting habitual shopping and eating with little hedonic focus; and taste-oriented (26.9%), who actively sought and enjoyed indulgent foods, sweets, and novelty. Taste-oriented consumers were disproportionately young adults (18–29 years) and city-dwellers.

### 3.3. TFEQ-R21: Eating Attitudes and Styles

#### 3.3.1. Descriptive Statistics

Descriptive statistics for all 16 TFEQ-R21 items are presented in Table 5 (4-point scale, recoded so that higher values indicate stronger endorsement). Emotional eating items had the lowest means (M = 1.79–1.88), so on average, respondents did not endorse them as self-descriptive. Uncontrolled eating items ranged from M = 1.96 to 2.29 and cognitive restriction items from M = 2.12 to 2.25, i.e., below the scale midpoint of 2.5, indicating neither strong endorsement nor clear rejection.

#### 3.3.2. Factor Analysis

EFA of the 16-item pool (maximum likelihood, Varimax rotation; KMO = 0.930; Bartlett’s *p* < 0.001) yielded the expected three-factor structure: emotional eating (six items; explained variance 42.9%; Cronbach’s α = 0.931; CR = 0.929), uncontrolled eating (seven items; 8.8%; α = 0.835; CR = 0.849), and cognitive restriction (three items; 5.4%; α = 0.750; CR = 0.626). The cumulative explained variance was 57.2%. CFA fit indices are given in Table 6: χ^2^/*df* was above 5 in both models, as is common with samples of this size, while CFI, TLI, NFI, and RMSEA were within conventional cut-offs. Szakály et al. [12] reported α = 0.927, 0.829, and 0.806. After recoding, the emotional eating factor showed a right-skewed distribution (skewness = +0.622), indicating that most respondents rejected these items as self-descriptive.

#### 3.3.3. Cluster Analysis

K-means cluster analysis yielded five distinct eating style segments (ANOVA; *p* < 0.01). Table 7 presents cluster sizes and proportions.

Indifferent (22.4%): No outliers on any eating attitude dimension. Cognitive workers and married adults are overrepresented. General low engagement with eating as a psychologically meaningful activity.

Uncontrolled Emotionless Eaters (16.2%): Strong rejection of emotional eating (the cluster scoring lowest on all emotional eating items), yet tendency toward uncontrolled eating patterns. Young men dominate. These consumers appear to eat in response to food cues rather than affect, a pattern linked to higher body weight in the literature (BMI by segment was not analysed).

Controlled Dieters (20.6%): Deliberate restriction is the defining feature. Emotional eating is entirely rejected. Members consciously limit intake for weight management, avoid fattening foods, and accept hunger as a consequence of self-discipline. Women over 60 years with higher education and strong health and environmental consciousness predominate. Widows and divorcees are overrepresented.

Elderly Uncontrolled Eaters (26.5%): The largest segment. Emotional eating is not characteristic; neither is self-regulation. Members simply eat until satiated without monitoring intake. Predominantly older, low-educated, and rural residents in Western and Southern Transdanubia. Income is insufficient for savings.

Uncontrolled Emotional Eaters (14.2%): The highest endorsement of both emotional eating and uncontrolled eating. Members eat in response to anxiety, sadness, tension, loneliness, and nervousness. Eating with others also triggers overeating. Predominantly older urban women in Central Hungary and South Transdanubia.

#### 3.3.4. Segment Perception of Functional Food Categories

Perceived palatability and perceived healthiness (each on a 1–5 scale) of 10 functional food categories were compared across the five TFEQ-R21 segments (Supplementary Tables S2 and S3). One-way ANOVA indicated differences (*p* < 0.05) in six of ten categories for palatability (mineral-enriched, probiotic, low-sugar, high-fibre, protein-enriched, and low-sodium products) and in eight of ten for healthiness (all except vitamin- and protein-enriched products). With a Bonferroni correction for 20 tests (*p* < 0.0025), four palatability differences (mineral-enriched, probiotic, high-fibre, and low-sodium products) and five healthiness differences (mineral-enriched, probiotic, low-fat, high-fibre, and gluten-free products) remained. Controlled Dieters gave the highest mean in eight of ten categories on each measure. The Indifferent segment gave the lowest palatability means for mineral-enriched, probiotic, low-fat, and high-fibre products, and Uncontrolled Emotionless Eaters for low-sugar, lactose-free, and low-sodium products. These unadjusted differences show that ratings differ across TFEQ-R21 segments; because the segments differ in age, sex, and education, and no adjusted models or pairwise tests were available, they do not show that segment membership predicts functional food reception.

### 3.4. Reliability Summary

Table 8 summarises reliability statistics across all three scales. Cronbach’s α exceeded 0.70 for all factors with three or more items, meeting accepted thresholds [41]. The two-item Habit- and Tradition-Driven Eating factor of the TEMS (α = 0.637) is below the usual 0.70 threshold and is interpreted cautiously. Composite reliability was low for Sceptical Disbelief (0.581), Belief in Energy Reduction (0.355), cognitive restriction (0.626), and Avoidance of Self-Indulgence (0.366); for the first two, CR is also hard to reconcile with the corresponding α values (0.705 and 0.738). These factors are treated as exploratory.

## 4. Discussion

This study provides a multi-dimensional profile of eating behaviour in the Hungarian adult population, using three validated instruments applied to nationally representative samples. The three scales point in a broadly similar direction: hedonic and habitual orientations and average profile segments were common, whereas health-oriented segments accounted for roughly 18–21% of respondents in each segmentation. These percentages depend on the clustering solution and the sample and should not be read as the population prevalence of health-conscious eating. This matches European surveys showing that unhealthy dietary patterns persist despite growing public health messaging [42,43]. It is also consistent with earlier Hungarian data showing that fewer than one in four adults make consciously health-driven food choices [12].

The TEMS factor analysis yielded eight factors, substantially fewer than the 15-factor German original [15] and the 17 factors reported by Phan and Chambers [16]. This is consistent with patterns seen in other non-German-speaking samples [17]. One possible, untested explanation is a more pragmatic, less differentiated motivational landscape in Hungarian food culture; the dominance of familiar, habitual motivations in the Traditional Simplifiers and Balanced Self-Rewarders segments is compatible with this. Because only 45 items were administered, the smaller number of factors may also reflect the reduced item set. Regarding H1, the original 15-factor structure was not reproduced, but this rests on EFA alone; a formal test (CFA of the original model or measurement invariance) was not performed. H2 was supported in that a health-oriented segment emerged, although cluster analysis always returns segments, so this is a weak test.

The HTAS results revealed a clear contradiction in Hungarian consumers’ thinking about health. Vitamin and mineral adequacy was rated as an important dietary goal, while cholesterol-raising foods were largely disregarded. This suggests that health attitudes are selective; however, food choices were not measured here, so we cannot say whether these attitudes match actual behaviour. This ambivalence is reflected in the relatively small Health-Conscious Metropolitan cluster (19.8%) relative to the much larger Misguided (30.4%) and Indifferent (26.1%) clusters. Similar patterns have been reported in other Central and Eastern European contexts [43], where stated health values do not translate into consistent food selection behaviour. The taste subscale cluster results echo this finding: the taste-oriented group (26.9%) and the Controversial Middle-Aged group (29.0%) together represent more than half the population with primarily hedonic food orientations.

The TFEQ-R21 results are broadly compatible with this picture: 20.6% of respondents formed the Controlled Dieters segment, while the other segments showed average, uncontrolled, or emotional eating profiles. The similar sizes of the health-oriented segments across instruments (18.3%, 19.8%, and 20.6%) should not be read as a stable minority of about 20%: the segments come from separate clustering solutions (the TEMS from a different sample), their sizes depend on the number of clusters chosen, and overlap in individual membership was not analysed. Cognitive restraint is also not the same as healthy eating, and with three weight-focused restriction items, the construct is narrow. The three-factor structure was supported in the 16-item model (H3), and a controlled-eating segment emerged (H4), consistent with Szakály et al. [12] and Szabó et al. [23]; as noted in the Introduction, H1–H4 were formulated with knowledge of earlier analyses of these data.

A new element of the present study is the comparison of functional food perceptions across TFEQ-R21 clusters. Controlled Dieters gave the highest mean palatability and healthiness rating in eight of ten categories each, but the ANOVA differences were not uniform across categories (Supplementary Tables S2 and S3). These unadjusted differences may be relevant to communication and product positioning, but they are cross-sectional, have not yet been adjusted for age, sex, and education and do not show that this segment is more receptive to dietary guidance. Uncontrolled Emotional Eaters rated healthiness higher than the Indifferent segment in nine of ten categories. Whether messaging aimed at this group would work better if it framed functional food consumption as a form of emotional self-regulation, rather than relying only on cognitive health benefit framing, remains a hypothesis for experimental testing.

Limitations. First, the data were collected in 2019 (TEMS) and 2021 (TFEQ-R21 and HTAS), and food behaviour has changed since the pandemic, so segment sizes may not describe the current population. Second, the TEMS and the TFEQ-R21/HTAS data come from independent samples collected before and during the COVID-19 pandemic; the segmentations, therefore, cannot be linked at the individual level, and differences between them may reflect period effects [44]. Third, quota sampling, face-to-face administration and self-reported attitudes, height, and weight may introduce selection and social desirability bias, and BMI, although collected, was not analysed by segment. Fourth, all instruments measure attitudes, motivations, and self-described eating styles, not actual intake. Fifth, the TFEQ-R21 was reduced from 21 to 16 items after EFA, as in [12] (the Hungarian adaptation [28] retained 21 items, and a 12-item version was reported in [23]); cognitive restriction retains only three weight-focused items, which narrows its content validity. The HTAS statements were formulated for this study and not taken from a separately validated Hungarian version, and the TEMS was administered with 45 items. Several factors have two items or low composite reliability, and discriminant validity (AVE, inter-factor correlations) is not documented. Sixth, EFA and CFA were run on the same sample with freed error covariances, and the CFA χ^2^/*df* values were above 5, so the fit indices are likely optimistic. Seventh, the cluster solutions are exploratory and were not tested for stability; segment profiles are descriptive and were not tested with corrected inferential statistics; and the functional food comparisons are unadjusted ANOVAs without pairwise tests.

Future research should examine whether health-oriented segments remain identifiable over time and replicate in new samples. It should also explore sustainability-related motivations, which are missing from the current TEMS version but have grown much more relevant to consumers [45], including in Hungary, where consumers increasingly report favourable beliefs and attitudes toward plant-based diets [46], and life-cycle-assessment-based modelling now allows the climate impact of food systems to be quantified and prioritised [47]. Longitudinal tracking of cluster membership and its association with dietary intake data and clinical health outcomes would further strengthen the practical validity of this framework.

## 5. Conclusions

Applying TEMS, HTAS, and TFEQ-R21 to two nationally representative samples of Hungarian adults shows that habitual, taste-oriented, and average-profile segments were common, while each instrument also yielded a smaller health-oriented segment (about 18–21%). Alongside them were groups characterised by uncontrolled or emotional eating attitudes or a focus on taste. These findings describe attitudes and self-reported motivations, not dietary behaviour, and segment sizes depend on the clustering solution.

These findings may inform the design of segment-specific communication, but this has not been tested. Whether health-framed messages mainly reach the health-oriented segments, and whether other segments respond to approaches built around convenience, habit change, emotional coping, or social norms should be examined in intervention and experimental studies. The differences in functional food perception across TFEQ-R21 segments are a starting point for such work, pending adjusted analyses and replication.

## Figures and Tables

**Table 1:** The 10 most important eating motivations (N = 1000).

‘I Eat What I Eat…’	Mean	SD	RSD (%)	Skewness
…because I love it.	6.01	1.360	22.63	−1.409
…because it tastes good.	5.87	1.423	24.24	−1.260
…because I know it well.	5.82	1.348	23.16	−1.177
…because I am hungry.	5.74	1.437	25.03	−1.130
…because that is what I am used to.	5.37	1.562	29.09	−0.823
…because that is what I usually consume.	5.24	1.621	30.93	−0.822
…because I enjoy eating it.	5.21	1.684	32.32	−0.780
…because it is quick to make.	5.11	1.584	30.99	−0.643
…because I think it is healthy.	5.11	1.639	32.07	−0.700
…because the food is appetizing.	5.10	1.671	32.76	−0.672

*SD, standard deviation; RSD, relative standard deviation.*

**Table 2:** The 10 least important eating motivations (N = 1000).

‘I Eat What I Eat…’	Mean	SD	RSD (%)	Skewness
…because it attracts me spontaneously (e.g., eye-level on shelves, attractive packaging).	3.01	1.830	60.79	0.380
…because I want to avoid disappointing someone who wanted to please me.	2.80	1.775	63.39	0.598
…because it would not be polite to others not to eat it.	2.67	1.751	65.58	0.667
…because it is familiar from commercials and I have seen it on television.	2.65	1.748	65.96	0.654
…because they expect me to eat it.	2.51	1.742	69.40	0.923
…because I want to appear good in front of others.	2.48	1.698	68.47	0.757
…because this food is trendy.	2.47	1.730	70.04	0.898
…because I feel lonely.	2.34	1.688	72.14	0.999
…because I am sad.	2.28	1.631	71.53	0.976
…because I am disappointed.	2.22	1.586	71.44	1.042

*SD, standard deviation; RSD, relative standard deviation.*

**Table 3:** TEMS consumer segments (N = 979 classified).

Cluster	n	%
Traditional Simplifiers	175	17.9
Frugal Social and Emotional Eaters	195	19.9
Balanced Self-Rewarders	270	27.5
Health-Conscious	179	18.3
Indifferent	160	16.4
Total (Classified)	979	100.0

**Table 4:** HTAS Health subscale consumer segments (N = 941 classified).

Cluster	n	%
Misguided Consumers	286	30.4
Health-Conscious Metropolitans	186	19.8
Health Risk Takers	223	23.7
Indifferent	246	26.1
Total	941	100.0

**Table 5:** Eating attitude statements: descriptive statistics (N = 1000).

Attitude Statement	Mean ^1^	SD	RSD (%)	Skewness
When I am depressed, I want to eat.	1.79	0.970	54.19	0.977
When I am lonely, I comfort myself by eating.	1.82	1.015	55.77	0.971
When I am nervous, I try to calm myself down by eating.	1.82	0.991	54.45	0.931
When I feel anxious, I start eating.	1.85	1.024	55.35	0.827
When I am sad, I often eat too much.	1.88	1.049	55.80	0.866
When I am tense or wound up, I often feel I need to eat.	1.88	1.003	53.35	0.840
When I start eating, sometimes I feel I cannot stop.	1.96	1.022	52.14	0.646
I am always so hungry that I find it hard to stop eating until I have finished everything on my plate.	1.97	0.998	50.66	0.688
I often feel a bottomless void in my stomach.	1.97	1.021	51.83	0.706
I am always hungry enough to eat at any time.	2.10	1.003	47.76	0.456
I do not eat certain foods because they are fattening.	2.12	1.095	51.65	0.504
When I see something delicious, I often get so hungry that I have to eat immediately.	2.19	0.986	45.02	0.366
I deliberately eat little at a time to keep my weight in check.	2.22	1.053	47.43	0.387
When I am with someone who is eating, it often makes me want to eat too.	2.25	1.024	45.51	0.298
I consciously limit how much I eat during meals to avoid gaining weight.	2.25	1.051	46.71	0.301
When I see or smell appetizing food, I find it very difficult not to eat, even if I have just finished eating.	2.29	0.970	42.36	0.171

*^1^ Scale: recoded for presentation so that 1 = ‘not at all true of me’ and 4 = ‘definitely true of me’ (higher = stronger endorsement). SD, standard deviation; RSD, relative standard deviation.*

**Table 6:** Confirmatory factor analysis fit indices.

Index	Acceptable Threshold	TFEQ-R21 Result	HTAS Health Result
CFI	>0.95	0.995	0.991
χ^2^/*df*	<5	5.05	10.29
TLI	>0.95	0.994	0.990
NFI	>0.95	0.994	0.987
χ^2^ (*df*)	–	510.4 (101)	1162.4 (113)
RMSEA	<0.06	0.054	0.049

*CFI, comparative fit index; χ^2^/*df*, chi-square to degrees of freedom ratio; TLI, Tucker–Lewis index (equivalent to the non-normed fit index); NFI, normed fit index; RMSEA, root mean square error of approximation.*

**Table 7:** TFEQ-R21 consumer segments (N = 976 classified).

Cluster	n	%
Indifferent	219	22.4
Uncontrolled, Emotionless Eaters	158	16.2
Controlled Dieters	201	20.6
Elderly Uncontrolled Eaters	259	26.5
Uncontrolled Emotional Eaters	139	14.2
Total	976	100.0

**Table 8:** Scale reliability: Cronbach’s α and composite reliability (CR) across all three instruments.

Scale/Factor	Cronbach α	CR
TEMS Factors		
Social Anxiety	0.916	—
Comfortable Enjoyment	0.876	—
Health Awareness & Weight Control	0.856	—
Price Sensitivity	0.782	—
Social Eating	0.800	—
Self-Reward	0.731	—
Habit- and Tradition-Driven Eating	0.637	—
Seductive Nature of Food	0.770	—
HTAS Factors		
Health Consciousness	0.837	0.878
Health Risk Behaviour	0.807	0.782
Sceptical Disbelief	0.705	0.581
Belief in Energy Reduction	0.738	0.355
TFEQ-R21 Factors		
Emotional Eating	0.931	0.929
Uncontrolled Eating	0.835	0.849
Cognitive Restriction	0.750	0.626

## Data Availability

The data presented in this study are available upon request from the corresponding author due to data ownership: the dataset was collected for the doctoral research of M.S. (University of Debrecen), who holds the data and releases them on reasonable request.

## Supplementary Materials

The following supporting information can be downloaded at: https://www.mdpi.com/article/10.3390/nu18213690/s1. Table S1: Weighted sample and population composition (Survey 1, TFEQ-R21 and HTAS); Table S2: Perceived palatability (1–5) of functional food categories by TFEQ-R21 segment (mean); Table S3: Perceived healthiness (1–5) of functional food categories by TFEQ-R21 segment (mean); Table S4: Confirmatory factor analysis results reported in the dissertation.

## Abbreviations

The following abbreviations are used in this manuscript: TFEQ-R21, Three-Factor Eating Questionnaire, Revised 21-item version; HTAS, Health and Taste Attitude Scales; TEMS, The Eating Motivation Survey; EFA, exploratory factor analysis; CFA, confirmatory factor analysis; PCA, principal component analysis; ANOVA, analysis of variance; BMI, body mass index; SD, standard deviation; RSD, relative standard deviation; CR, composite reliability. AVE, average variance extracted; CFI, comparative fit index; RMSEA, root mean square error of approximation; SRMR, standardised root mean square residual; TLI, Tucker–Lewis index.
