# Supplementary material for: Multi-Dimensional Assessment of Eating Behaviour in the Hungarian Adult Population: Motivations, Health–Taste Attitudes, and Eating Styles Using TEMS, HTAS, and TFEQ-R21
Source: Nutrients. 2026 Nov 7;18(21):3690. doi: 10.3390/nu18213690 (PMC13765390; doi:10.3390/nu18213690)
Supplement: Supplementary file 1 [file nutrients-18-03690-s001.zip › nutrients-4620866-supplementary.pdf]

Supplementary Tables

All values are taken from the doctoral dissertation of M.Z.S. (University of Debrecen, 2025). Yellow text marks points the authors must check.

Table S1. Weighted sample and population composition (Survey 1, TFEQ-R21 and HTAS)

Characteristic	Weighted sample, %	HCSO population, %
Sex: men	47.1	47.8
Sex: women	52.9	52.2
Age 18–29	17.2	17.2
Age 30–39	16.1	16.0
Age 40–49	19.6	19.6
Age 50–59	15.1	15.1
Age 60 or older	32.1	32.1
Capital	18.4	17.9
Other town	54.5	52.6
Village	27.1	29.5
Central Hungary	31.0	31.0
Central Transdanubia	10.8	10.8
Western Transdanubia	10.2	10.1
Southern Transdanubia	9.1	9.0
Northern Hungary	11.4	11.5
Northern Great Plain	14.7	14.8
Southern Great Plain	12.8	12.7
Education: max. 8 years of primary school	10.8	n/a
Education: vocational	30.3	n/a
Education: secondary school leaving	41.7	n/a
Education: tertiary	16.8	n/a
Education: no answer	0.3	n/a

Maximum deviation from the population: 2.4 percentage points (villages). Four typographical errors in the dissertation table were corrected (men 41.1% to 47.1%; 161, 147 and 128 entered as percentages for 16.1%, 14.7% and 12.8%). The unweighted sample composition is not available.

Table S2. Perceived palatability (1–5) of functional food categories by TFEQ-R21 segment (mean)

Functional food category	Indifferent	Uncontrolled Emotionless	Controlled Dieters	Elderly Uncontrolled	Uncontrolled Emotional	ANOVA p
Vitamin-enriched (e.g., muesli, soft drinks)	3.78	3.76	3.82	3.70	3.76	0.843
Mineral-enriched (e.g., calcium-enriched cheese, milk)	3.64	4.33	4.35	4.16	4.28	<0.001
Probiotic (e.g., yogurt, kefir, margarine)	3.64	4.12	4.28	3.94	4.14	<0.001
Low-sugar or sugar-free	3.32	3.19	3.59	3.40	3.43	0.017
Low-fat	3.45	3.57	3.72	3.46	3.53	0.061
Lactose-free	3.36	3.13	3.49	3.28	3.17	0.068
High-fibre (e.g., fibre-enriched drinks)	3.81	4.37	4.29	4.03	4.06	<0.001
Gluten-free (e.g., bread)	3.46	3.30	3.41	3.34	3.27	0.530
Protein-enriched (e.g., energy bars)	3.49	3.22	3.54	3.33	3.22	0.043
Low-sodium	3.35	2.85	3.37	3.24	3.20	<0.001

One-way ANOVA p-values as reported in the dissertation (shown as <0.001 where reported as 0.000). With 20 tests, a Bonferroni threshold is 0.0025. SDs, F values, effect sizes and pairwise tests are not available.

Table S3. Perceived healthiness (1–5) of functional food categories by TFEQ-R21 segment (mean)

Functional food category	Indifferent	Uncontrolled Emotionless	Controlled Dieters	Elderly Uncontrolled	Uncontrolled Emotional	ANOVA p
Vitamin-enriched (e.g., muesli, soft drinks)	3.97	3.85	3.92	3.87	4.04	0.461
Mineral-enriched (e.g., calcium-enriched cheese, milk)	3.81	4.52	4.39	4.29	4.29	<0.001
Probiotic (e.g., yogurt, kefir, margarine)	3.85	4.37	4.43	4.24	4.30	<0.001
Low-sugar or sugar-free	3.89	4.01	4.22	4.02	4.18	0.005
Low-fat	3.87	4.08	4.30	4.04	4.17	<0.001
Lactose-free	3.88	4.05	4.22	3.97	4.06	0.017
High-fibre (e.g., fibre-enriched drinks)	3.87	4.34	4.38	4.20	4.19	<0.001
Gluten-free (e.g., bread)	3.85	3.99	4.24	4.05	4.08	0.002
Protein-enriched (e.g., energy bars)	3.80	3.73	3.87	3.77	3.60	0.263
Low-sodium	3.78	3.94	4.13	4.03	3.97	0.009

Table S4. Confirmatory factor analysis results reported in the dissertation

Index	TFEQ-R21 (16 items)	HTAS health (17 items)	HTAS taste (16 items)
χ^2	510.389	1162.365	1224.993
df	101	113	94
χ^2/df	5.05	10.29	13.03
CFI	0.995	0.991	0.992
GFI	0.995	0.990	0.993
TLI	0.994	0.990	0.990
NFI	0.994	0.987	0.991
NNFI	0.994	0.965	0.990
RMSEA	0.054	0.049	0.032

Error covariances between items of the same latent variable were allowed where modification indices were large. SRMR was not reported.
